# Effects of 1-Methylcyclopropene Treatment on Postharvest Quality and Metabolism of Different Kiwifruit Varieties

**DOI:** 10.3390/foods13223632

**Published:** 2024-11-14

**Authors:** Yanni Zhao, Meiru Yan, Kun Zhang, Xuan Wu, Zi Wang, Ting Shao, Jing Lei, Xuefeng Chen, Huan Liu

**Affiliations:** 1 School of Food Science and Engineering, Shaanxi University of Science & Technology, Xi’an 710021, China; 2 Shaanxi Research Institute of Agricultural Products Processing Technology, Xi’an 710021, China; 3 Shaanxi Rural Science and Technology Development Center, Xi’an 710054, China

**Keywords:** kiwifruit, 1-MCP, storage quality, metabolomics

## Abstract

The kiwifruit (*Actinidia*) is an important nutritional and economic fruit crop. However, the short edible window period of kiwifruit has seriously affected its market value. 1-Methylcyclopropene (1-MCP), as a novel ethylene inhibitor, is widely applied to delay fruit ripening and senescence. To our knowledge, there are limited studies on the effects of 1-MCP on fruit quality and metabolism of different kiwifruit varieties. Three kiwifruit cultivars (i.e., ‘Xuxiang’, ‘Huayou’, and ‘Hayward’) widely cultivated in China were chosen as our research objects. The variations of storage quality and metabolic characteristics of kiwifruits treated with various 1-MCP concentration (0 μL/L, 0.5 μL/L, and 1.0 μL/L) were systematically investigated. The results showed that 1-MCP treatment significantly improved the quality of kiwifruit during storage. Among them, for ‘Xuxiang’ and ‘Hayward’ varieties, 1.0 μL/L 1-MCP treatment could delay the decrease in fruit firmness, the increase in maturity index and cellulase activity, and inhibit the decrease in ascorbic acid (AsA) level. However, the 0.5 μL/L 1-MCP had a great influence on the chlorophyll content and maturity index of the ‘Huayou’ cultivar, and the preservation effect was satisfactory. In addition, gas chromatography–mass spectrometry (GC–MS) based metabolomics studies revealed that 1-MCP treatment affected carbohydrates metabolism, fatty acids metabolism, and amino acids metabolism in different kiwifruit varieties. Correlation analysis indicated that sugars metabolism has the closest relationship with postharvest physiological quality. This research indicated that the effectiveness of 1-MCP treatments was dependent on fruit variety and treatment concentration. Furthermore, these findings provide a theoretical foundation for extending the shelf life of different kiwifruit varieties.

## 1. Introduction

Kiwifruit has extremely high nutritional and medicinal value, containing health-enhancing compounds and serving as a good source of calcium, potassium, tannins, and various amino acids [1]. Currently, there are 75 reported taxa of kiwifruit, including 54 species and 21 varieties in the world [2]. China, as the world’s largest kiwifruit-growing country, has abundant kiwifruit germplasm resources [3]. With the rapid development of the kiwi industry, certain kiwi varieties have achieved greater competitive edge and have become some of the most important commercial members in the international fruit trade. Among them, the ‘Xuxiang’ kiwifruit, originating from Xuzhou City in Jiangsu Province of China, is known for its strong adaptability, early fruiting, and delicious sweetness, making it well received by consumers [4]. ‘Huayou’, a variety bred from the indigenous kiwifruit of Shaanxi, is a natural hybrid of *Actinidia chinensis* and *Actinidia deliciosa*, with exceptional advantages in terms of yield, quality, flavor, resistance, and shelf life [5]. ‘Hayward’ has greater weight and higher soluble solids contents than other kiwifruit varieties, and the fruit is hard enough to withstand physical and temperature management during commercial handling and storage [6,7], which has dominated the international market for many countries (e.g., Italy, New Zealand, and China) [8]. However, kiwifruit is a respiratory leapfrog fruit, and most kiwifruit varieties soften rapidly after harvest, deteriorate progressively as mature, or lose their food value due to infection by pathogens, all of which significantly limit their market potential [9]. Therefore, research on postharvest storage of kiwifruits will play an important role in maintaining kiwifruit quality during long-term storage and transport in the future.

Many researchers have attempted to prolong the storage time of kiwifruit through exogenous treatment, including refrigeration [10], edible packaging materials [11,12], and gas fumigation techniques [13,14]. Previous studies have shown that low temperature is a convenient and effective method for extending the shelf life of kiwifruit. However, low temperatures can trigger physiological disorders in kiwifruit, leading to significant commercial risks [15]. Edible packaging materials are efficacious in preserving food quality and extending shelf life by hindering microbial spoilage and providing moisture and gas barrier properties [16]. Nonetheless, these materials are currently facing challenges in terms of practical acceptance due to the uncertainty surrounding their potential impacts on consumer health [17]. Gas fumigation technology is a promising method to enhance fruit quality postharvest, contributing to delaying senescence, preventing browning, managing diseases, and alleviating chilling effects [18]. Among these technologies, 1-MCP is highlighted as a key fumigation preservation technique that helps mitigate the impacts of external and internal ethylene, thereby extending the shelf life of fruit by regulating respiratory processes [19]. The widespread applications of 1-MCP in fruit and vegetable storage and preservation are attributed to its nontoxic, odorless, highly efficient, stable, safe, and environmentally friendly characteristics [20]. The effect of 1-MCP is mediated by blocking the ethylene perception in plant tissues [21]. Ethylene has the capability to coordinate with electrons within the metal ions of its receptor proteins, whereas the ability of 1-MCP to retrieve electrons from metals is stronger than that of ethylene, and, thus, 1-MCP irreversibly binds strongly with functional ethylene receptor proteins during competition for the binding site, consequently impacting ethylene signaling [22,23]. Numerous studies have been conducted to investigate the efficacy of 1-MCP treatment in mitigating fruit dehydration and shrinkage, pulp softening, spoilage, and mildew [24,25,26]. Plums treated with 1.2 μL/L of 1-MCP exhibited elevated firmness and decreased amounts of cell-wall-degrading enzymes (i.e., pectinesterase, polygalacturonase, cellulase, and β-galactosidase), resulting in a reduction in the degradation of cell wall polysaccharides and subsequently retarding the softening progression in plums [27]. Pre- or postharvest applications of 1-MCP also produced potential metabolic alterations in apple fruit, which might directly suppress ethylene metabolism or influence stress responses and signaling pathways indirectly [28]. By preserving the amounts of nonenzymatic antioxidants and boosting the activity of reactive oxygen species (ROS)-scavenging enzymes, 1-MCP and modified atmosphere packaging (MAP) therapy can effectively stop figs from deteriorating, softening, and losing weight [29]. In kiwifruit, 1.0 μL/L 1-MCP treatment significantly inhibited the respiration rate, delayed the decrease in fruit firmness, and increased the soluble solids content (SSC) in ‘Jinyan’ kiwifruit [30]. Concurrently, 1-MCP, as a prestorage treatment in kiwifruit, could effectively reduce fruit softening and rotting due to low ethylene production and wound, resulting in significant increases in total soluble phenolics, flavonoids, and antioxidant capacity during the treatment period [31]. In addition, differences in internal ethylene concentration and ethylene receptor sites between different cultivars of fruits can further modulate the preservation and regulatory role of 1-MCP on the fruit ripening ability [32], indicating that the efficacy of 1-MCP treatment on fruits is influenced by different cultivars, as evidenced in previous research on apples [33], pomegranates [34], and apricots [35].

The varying concentrations of 1-MCP treatment will result in distinct metabolic traits during fruit ripening, particularly pronounced when considering the substantial flavor variances among different kiwifruit cultivars. Thus, the impacts of 1-MCP treatment on the quality and metabolism of various kiwifruit varieties during storage merit further investigation. The primary objectives of our study were to assess the specific efficacy of 1-MCP in preserving the storage quality of the major kiwifruit cultivars (i.e., ‘Xuxiang’, ‘Huayou’, and ‘Hayward’) in Shaanxi Province of China, alongside considering the alterations in kiwifruit metabolism following 1-MCP treatment, and to investigate the relationship between the kiwifruit metabolic characteristics and quality parameters after 1-MCP treatment.

## 2. Materials and Methods

### 2.1. Chemicals

In this study, 1-MCP (Xianyang Xiqin Biotechnology Co., Ltd., Xianyang, China), phenolphthalein (Tianjin Tianli Chemical Reagents Co., Ltd., Tianjin, China), sodium hydroxide, quartz sand, and ferrous chloride (Xianshuigu Industrial Company, Tianjin, China), oxalic acid, 2, 6-dichlorophenol indophenol, phenol, acetone, 30% hydrogen peroxide, methanol, citrate, calcium carbonate, potassium persulfate, sodium dihydrogen phosphate, ferrous sulfate AsA, copper sulphate, ferrous chloride, and ethanol (Tianjin Kermel Chemical Reagent Co., Ltd., Tianjin, China), sodium chloride, ethylenediaminetetraacetic acid (EDTA), polyvinylpyrrolidone (PVP), carboxymethylcellulose (CMC), 3,5-dinitrosalicylic acid, potassium sodium tartrate, ABTS, DPPH, pyrocatechol violet, and ferrozine monosodium salt (Shanghai Ruiyong Biotechnology Co., Ltd., Shanghai, China) were used. The reagents used in metabolomics analysis including methoxyamine hydrochloride, pyridine, and N-methyl-N-(trimethylsilyl) trifluoroacetamide (MSTFA) (Sigma-Aldrich, St. Louis, MO, USA). The standard chemicals for identification and validation (Alfa Aesar, Heysham, UK), Sigma-Aldrich, J&K Scientific (Beijing, China).

### 2.2. Plant Material and Postharvest Treatments

Three kiwifruit cultivars (i.e., ‘Xuxiang’, ‘Huayou’, and ‘Hayward’) were harvested from the Shaanxi Bairui Kiwifruit Research Institute, Xi’an, China in September–October 2020 at maturity stage SSC of approximately 5.5–6.5%. The kiwifruits, which were uniform and free of defects and mechanical damage, were transported to the laboratory on the same day of harvest and left overnight to eliminate field heat. The kiwifruits were then sealed in boxes wrapped with polyvinyl chloride film and filled with 1-MCP (0, 0.5, and 1.0 μL/L) and kept at 20 °C for 24 h. Following these treatments, the kiwifruits were shifted out and stored in an incubator at a constant temperature of 20 °C (MGC-400HP, Shanghai, China). Different kiwifruit varieties were analyzed at three-day intervals over a period of 15 d to monitor the quality changes.

### 2.3. Kiwifruit Quality Analyses

#### 2.3.1. Determinations of Firmness, Chlorophyll, and Browning Index (BI)

Fruit firmness was measured at four equally spaced points using a physical property analyzer (TA-TX 2i, Godalming, UK) with a P/2 probe, and the measuring results were recorded according to the maximum peak value of the probe when it first penetrated the equatorial part of the fruit 1 cm at a speed of 1 mm/s. Each group received the same treatment three times in parallel for all three kiwifruits.

Chlorophyll content was detected by a visible spectrophotometer (WFJ7200, Shanghai, China). Fresh samples (1 g) were mixed with a small amount of quartz sand and calcium carbonate powder, along with 2–3 mL 80% acetone. The mixture was milled into a uniform pulp, after which 10 mL of acetone was added, and grinding persisted until the tissue turned white. The solution was allowed to settle for 3–5 min, then filtered, before fixing the volume to a 50 mL volumetric flask. Subsequently, absorbance values of kiwifruit extracts were determined at wavelengths of 663 nm and 645 nm. As a blank control, an 80% acetone solution was used. Chlorophyll a, b, and total chlorophyll content were calculated using the equations below.

(1) $$\mathrm{Chlorophyll}\;a\;\mathrm{content}:\;C_{a}=\frac{(12.7{\;A}_{663\mathrm{nm}}-2.69\;A_{645\mathrm{nm}})\;\times \;V}{1000\;W}$$

(2) $$\mathrm{Chlorophyll}\;b\;\mathrm{content}:\;C_{b}=\frac{(22.9\;A_{645\mathrm{nm}\;}-\;4.68\;A_{663\mathrm{nm}})\;\times \;V}{1000\;W}$$

(3) $$\mathrm{Chlorophyll}\;\mathrm{total}\;\mathrm{content}{:\;C}_{a+b}={\;C}_{a}\;{+C}_{b}$$

In the formula, A_663nm_ and A_645nm_: the absorbances of the samples at the wavelength A_663nm_ and A_645nm_, respectively; V: the volume of the extract; W: the weight of the sample.

A spectrocolorimeter (CM-5, Tokyo, Japan) was employed to observe the a*, b*, and L* values of the color of the kiwifruit cross section, and the BI was calculated according to the equations below.

(4) $$x=\frac{a+1.74\;L}{5.645\;L+a-3.012\;b}$$

(5) $$\mathrm{BI}=\frac{100\;\times \;(x-0.31)}{0.172}+180$$

#### 2.3.2. Analyses of Titratable Acid (TA), SSC, Reducing Sugar, and Cellulase Activity

TA content was detected by acid–base titration; 5 g of the sample was homogenized with water and transferred to a 50 mL volumetric flask, followed by filtration. Subsequently, 10 mL of the sample solution was titrated with 0.1 mol/L NaOH standard solution using phenolphthalein as the indicator, and the end point of the titration appeared pink color and lasted for 2 min [36]. For the determination of SSC content, 1 g kiwifruit sample was ground into a homogenate, centrifuged at 10,000 rpm for 20 min, and the supernatant was collected and analyzed using an Abbe refractometer (2W, Shanghai, China). Maturity index (SSC/TA) was expressed as the ratio of SSC to TA. The reducing sugar content of kiwifruit was determined by referring to the recent method of Zhang et al. [37].

For cellulase activity assay, kiwifruit samples (2 g) were homogenized with 6 mL of enzyme extract (6% NaCl, with 0.6% EDTA, 1% PVP) and centrifuged at 10,000 rpm for 20 min using a Cence centrifuge (H-1850R, Hunan, China). The supernatant was considered as the crude enzyme extract. To initiate the enzymatic reaction, 0.1 mL of a diluted 1/10 enzyme extract was transferred to 1.0 mL of carboxymethylcellulose solution (pH 4.0), and 1.0 mL of citrate buffer (pH 4.0) was used as a control. The reaction took place at a consistent temperature of 40 °C for a duration of 30 min. Subsequently, 0.9 mL of 3,5-dinitrosalicylic acid (DNS) reagent was introduced, followed by a 5 min incubation in a boiling water bath. The final volume was adjusted to 15 mL with water and thoroughly mixed. Absorbance readings were taken at 540 nm. Cellulase activity units (U) were quantified as 1 μg of glucose per gram of fresh sample per minute of carboxymethyl cellulose (CMC) breakdown.

#### 2.3.3. AsA, ·OH, and ABTS ^•+^ Assays

The content of AsA was quantified through the 2,6-dichloroindophenol titration method [38] with some modifications. The sample was subjected to homogenization using an oxalic acid solution and subsequently titrated with 2,6-dichloroindophenol reagent until a light pink color was achieved, which remained stable for at least 15 s. Each set of samples was titrated three times and a blank titration was performed. According to the formula described by Nerdy [38], AsA levels were calculated and presented as mg/100 g fresh weight (FW).

The kiwifruit samples were homogenized and centrifuged at 14,000 rpm for 10 min to obtain the supernatant. The supernatant, diluted 10 times, was used for the determination of ·OH and ABTS^•+^ analyses.

The ·OH radical scavenging capacities assay was conducted using the previous method [39] with slight modifications. Firstly, both 1 mL of FeSO_4_ (9 mmol/L) and 1 mL salicylic acid–ethanol (9 mmol/L) were fully mixed with 1 mL kiwifruit sample diluent and 1 mL H_2_O_2_ (8.8 mmol/L). Subsequently, the mixture was incubated for 20 min in a 37 °C water bath, and then the absorbance (As) was recorded at 510 nm. A_C_ was the absorbance of water as the blank control.

ABTS^•+^ radical scavenging capacities were estimated using the method described by the previous work [40] with some modifications. The ABTS^•+^ solution was produced by reacting 0.17 mmol/L potassium persulfate (K_2_S_2_O_8_) and 7.00 mmol/L ABTS, and storing in the dark for 16 h at room temperature. The ABTS^•+^ solution was diluted in phosphate buffer (pH = 7.4) until the absorbance value of 0.7 ± 0.02 at 734 nm. Afterwards, 30 μL kiwifruit sample diluent was thoroughly mixed with 2.97 mL of the ABTS^•+^ solution, and the absorbance value (As) was measured at 734 nm following a 10 min reaction. The absorbance (Ac) was measured using water as a blank control.

The free radical scavenging rate was calculated using the following formula (6). Each group received the same treatment three times in parallel for all three kiwifruits.

(6) $$\mathrm{Scavenging}\;\mathrm{capacities}=(1-\frac{As}{\mathrm{Ac}})\;\times \;100\%$$

### 2.4. Metabolomics Analysis

To investigate the effects of 1-MCP on the kiwifruit variety characteristics throughout different storage durations, samples from three kiwifruit cultivars (i.e., ‘Xuxiang’ ‘Huayou’, and ‘Hayward’) were collected at 0 d, 6 d, and 15 d after exposure to different levels of 1-MCP (0, 0.5, and 1.0 μL/L). Kiwifruits were quickly peeled, seeded, and broken by a juicer and then vacuum dried to powder using a freeze dryer (CTFD-10S, Shandong, China). Subsequently, 100 mg powder was weighed and immersed in 1.5 mL of 80% methanol, and the vortexed mixing method was employed for 10 min to disrupt the plant cell wall and extract endogenous metabolites. After being centrifuged at 14,000 rpm for 10 min, 500 µL of the supernatant was concentrated using a vacuum refrigerated concentrator (Labconco, Kansas City, MO, USA). Following this treatment, 140 µL of methoxyamine solution (20 mg/ mL in pyridine) was added into the dried specimen, vortexed 3 min until the sample was fully dissolved, and then incubated in a 37 °C water bath for 90 min. After that, 30 µL of MSTFA was added to the mixture and then derivatized for 60 min in a 37 ℃ water bath. Finally, 80 μL of the supernatant was collected in an injection vial for GC–MS-TQ8040NX (Shimadzu, Kyoto, Japan) analysis.

The separation of sample was carried out using an Agilent DB-5 MS capillary column (30 m × 0.25 mm × 0.25 μm). High-purity helium (99.9995%) served as the carrier gas, with a flow rate of 1.2 mL/min under constant flow mode. The temperature of injection was adjusted to 300 °C, with a 1 µL sample injected into the column at a 20:1 split ratio. The oven temperature program ranged from 70 °C for 3 min to 310 °C, maintained for 5 min at the rate of 10 °C/min. The ion source temperature was maintained at 230 °C, and metabolite ionization was performed in electron ionization mode with 70 eV. The solvent delay time was 3.5 min and the mass scan range was 33–600 *m*/*z* with a 0.25 scans s^−1^ scan speed. The metabolome analysis was conducted six times, with quality control (QC) samples systematically incorporated throughout the assay to ensure the integrity of the analytical process.

### 2.5. Data Processing and Statistical Analysis

Firstly, the metabolic profile data of the QC sample were imported into the ChromaTOF version 4.72.0.0 (LECO, Powell, TN, USA) and AMDIS version 2.70 (NIST, Gaithersburg, MD, USA) software programs for deconvolution and identification of peaks. Secondly, metabolites were identified through comparison with commercial standard mass spectral libraries (NIST 17, Wiley, Fiehn, etc.), followed by verification of qualitative results using retention time (RT), retention indices (RI), and mass spectral features of standard samples. The established quantitative table containing characteristic ions and the corresponding RT of metabolic features was imported into the GC–MS Postrun analysis workstation for peak alignment and metabolites integration. The peak areas of the metabolites were normalized to the total areas for subsequent data analysis.

An overview analysis of the metabolic distinctions of various kiwifruit cultivars over the storage period following exposure to different levels of 1-MCP was performed, employing partial least squares discriminant analysis (PLS-DA) in SIMCA-P 11.0 software (Umetrics, Umeá, Sweden). Two-way ANOVA and metabolic pathway enrichment analyses were completed using MetaboAnalyst (http://www.metaboanalyst.ca/ accessed on 4 June 2024). Cluster analysis and correlation network analysis of physiological traits and differential metabolites were conducted utilizing Chiplot (https://www.chiplot.online/ accessed on 4 June 2024). Univariate statistical analysis (*p* < 0.05) was performed using IBM SPSS Statistics 23.0 (Statistical Package for the Social Sciences). The visualization of physiological indicators and Supplementary Data was accomplished using Origin 2022 and Adobe Illustrator CC 2018.

## 3. Results and Discussion

### 3.1. Effects of 1-MCP Treatment on Qualities Characteristics of Kiwifruits

#### 3.1.1. Impacts of 1-MCP Treatment on Postharvest Sensory Qualities of Kiwifruits

**Figure 1:**
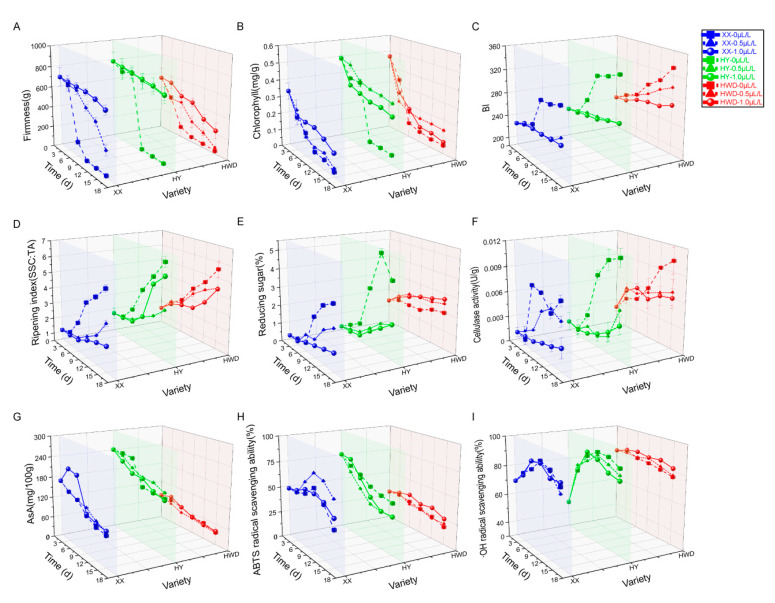
Effects of 1-MCP treatment on postharvest physiological qualities of kiwifruits. (**A**) firmness, (**B**) chlorophyll content, (**C**) browning index, (**D**) maturity index (SSC/TA), (**E**) reducing sugar content, (**F**) cellulase activity, (**G**) ascorbic acid content, (**H**) ABTS^•+^ free radical scavenging capacity, (**I**) ·OH free radical scavenging ability. XX, HY, and HWD represent ‘Xuxiang’, ‘Huayou’, and ‘Hayward’, respectively.

The firmness of kiwifruit plays an important role in the assessment of its sensory qualities, and is significantly related to fruit acceptability [41]. Kiwifruit, as a climacteric fruit, softens primarily attributed to the accumulation of excess ethylene [42]; thus, the decrease in firmness can be alleviated by applying 1-MCP to inhibit the plant’s ethylene response. In our study, the kiwifruit firmness in the 1-MCP untreated (0 μL/L 1-MCP) group significantly decreased along with the extension of storage time, reaching a low level at 9 d. In contrast, the decrease rate of kiwifruit firmness in the 1-MCP-treated group (0.5 and 1.0 μL/L 1-MCP) gradually diminished along with the storage time extended. Specifically, for both ‘Xuxiang’ and ‘Hayward’ varieties, the decreasing trend of fruit firmness diminished as the concentration of 1-MCP treatment increased; therefore, after treatment with 1.0 μL/L 1MCP, the decline in fruit firmness was the slowest. In contrast, there were minimal differences in ‘Huayou’ firmness after applying various concentrations of 1-MCP treatments (0.5 and 1.0 μL/L 1-1MCP), suggesting that the 0.5 μL/L 1-MCP treatment could be the optimal concentration in delaying the firmness reduction in ‘Huayou’ kiwifruit (Figure 1A). The delayed impacts of 1-MCP treatment on the firmness reduction in different kiwifruit varieties could be attributed to variations in receptor sensitivity of various fruits during ripening, because the availability and regeneration capacity of ethylene binding sites following 1-MCP treatment vary among different fruit varieties, even within the same species [43].

Chlorophyll serves a primary function for plant photosynthesis, and its degradation is related to the metabolic processes associated with ethylene-induced senescence [44]. Some studies have found that 1-MCP treatment could alter the promoter activity of chlorophyll-related genes, selectively repress genes involved in chlorophyll degradation, or inhibit ethylene synthesis genes and signal transduction [45,46,47] to directly or indirectly affect the chlorophyll biosynthesis of the fruit. In our study, the chlorophyll levels in kiwifruit displayed a consistent decline with the extension of storage time. Furthermore, the impact of 1-MCP treatment on chlorophyll level significantly varied among different kiwifruit varieties during storage. Specifically, 1-MCP displayed less effect on the chlorophyll levels in ‘Xuxiang’ and ‘Hayward’ before 6 d of storage, but a notable delay in the decline of chlorophyll content was noticed after 1.0 μL/L 1-MCP treatment in ‘Xuxiang’ and 0.5 μL/L 1-MCP treatment in ‘Hayward’ for 9 d (Figure 1B). On the 15th day of storage, compared to the control group, the chlorophyll levels in ‘Huayou’ and ‘Hayward’ treated with 0.5 μL/L 1-MCP decreased from 88.54% and 89.30% to 28.93% and 69.90%, respectively. Meanwhile, ‘Xuxiang’ treated with 1.0 μL/L 1-MCP experienced a reduction in chlorophyll content from 91.07% to 59.42% (Supplementary Figure S1). In the untreated group of 1-MCP, the chlorophyll degradation rates of the three kiwifruit varieties were not uniform. Among them, ‘Huayou’ exhibited the fastest decrease in chlorophyll level, while ‘Xuxiang’ had lower chlorophyll levels compared to ‘Huayou’ and ‘Hayward’ at harvest. Furthermore, the variation in chlorophyll level in the ‘Huayou’ showed the greatest sensitivity to 1-MCP treatment (Figure 1B). These results may be closely related to the differences in the biosynthesis, degradation, or turnover of chlorophyll in different kiwifruit varieties [48].

As fruit ripening progresses, the acceleration of the senescence process results in the initiation of browning, causing a quicker deterioration of quality and a shorter shelf life [49]. Reduced fruit energy levels with prolonged storage time could also lead to increased susceptibility of the pulp to browning [50], while 1-MCP treatment may prevent pulp browning by extending the senescence process, decreasing the overall accumulation of phenolics and phenylalanine deaminase activity [51]. After the storage time reached 6 d in our study, BI of different kiwifruit varieties samples gradually increased over time; among them, the BI values of the control groups of ‘Xuxiang’ and ‘Huayou’ sharply increased during the 6–9 d, but this upward trend was significantly suppressed after treatment with 0.5 μL/L and 1.0 μL/L 1-MCP (Figure 1C). The BI value of ‘Xuxiang’ in the untreated group increased by 25.10% at 15 d compared with that at 0 d, whereas the rates of increase in BI value for 0.5 μL/L and 1.0 μL/L 1-MCP treatment groups became 9.89% and 5.46% (Supplementary Figure S1). For ‘Huayou’, the increase rate of BI value in the groups treated with 0.5 μL/L and 1.0 μL/L 1-MCP compared to the untreated group decreased from 29.53% to 7.92% and 7.85%, respectively, at 15 d (Supplementary Figure S1). Similarly, the increase rate of BI values in the ‘Hayward’ for the untreated group compared to the groups treated with 0.5 μL/L and 1.0 μL/L 1-MCP at 15 d decreased from 26.97% to 18.97% and 9.98%, respectively (Supplementary Figure S1). These results indicate that the 1-MCP can effectively improve the color deterioration in kiwifruit flesh, with a positive correlation between the 1-MCP concentration level and its efficacy.

#### 3.1.2. Effects of 1-MCP Treatment on Postharvest Flavor Characteristics of Kiwifruits

The conversion of insoluble starch to SSC is one of the crucial features of kiwifruit ripening, and the TA level is closely related to the fruit flavor [52]. Therefore, maturity index (SSC/TA) is traditionally utilized for assessing the ripeness of the harvest [53]. During storage in our study, the kiwifruit SSC exhibited a gradual increase, while the TA content showed a gradual decrease over time (Supplementary Figure S2). Consequently, the maturity index (SSC/TA) exhibited a gradual increase with the duration of storage. In Figure 1D, the maturity index of fruits treated with 1-MCP increased at a slower rate compared to the untreated group, and the maturity index of the 1-MCP-treated group was significantly lower than that of the control group across all varieties after 9 d of storage. The response of ‘Xuxiang’ and ‘Hayward’ to 1-MCP increased in a concentration-dependent pattern, whereas the optimal treatment concentration for ‘Huayou’ was 0.5 μL/L, proving more effective than 1.0 μL/L treatment. These results suggested that 1-MCP treatment was effective in inhibiting the increase in SSC and delaying the reduction in TA content, thereby extending the kiwifruit ‘s desirable eating quality. Similar results were observed in a previous study on the softening of ‘Cuixiang’ versus ‘Hayward’ kiwifruits under refrigeration conditions [54]. Different cultivars did not exhibit a uniform increase in maturity index over extended storage durations, which may be affected by the influx of soluble carbohydrates or the disintegration of starch [55].

Reducing sugars are the main soluble carbohydrates in most fruits [56]. In various studies focusing on respiratory-leap fruits, such as peach and apple [57,58], the accumulation rate of reducing sugar content in the fruit was retarded by 1-MCP treatment, leading to a prolonged shelf life. Our study also observed that 1-MCP treatment decelerated the increasing trend of reducing sugar content in kiwifruit. Within 15 d of storage, the reducing sugar levels of ‘Xuxiang’ and ‘Huayou’ started to increase sharply after 6 d. The applications of 1-MCP significantly slowed down this trend, and a higher concentration of 1-MCP resulted in a more effective inhibition of the rise in reducing sugar content (Figure 1E). In addition, the untreated group of ‘Huayou’ showed the most significant increase in reducing sugar level during storage. This observation may indicate that ‘Huayou’ has a shorter consumption window compared to the other two varieties (i.e., ‘Xuxiang’ and ‘Hayward’), as fruits with higher soluble sugar accumulation tend to be sweeter and reach maturity earlier [59]. Furthermore, the application of 1-MCP treatment appeared to have a contradictory impact on the reducing sugar levels in ‘Hayward’ kiwifruit. The group of fruits treated with 1-MCP exhibited higher levels of reducing sugars compared to the control group throughout the storage period. This finding aligned with a previous study on 1-MCP-treated ‘Hayward’ kiwifruit [31] The observed phenomenon may be attributed to the softening pattern of ‘Hayward’ kiwifruit, which potentially helped in preserving favorable fruit storage qualities and mitigating the negative effects of reducing sugar accumulation [60].

The changes in the content and ratio of sugars and starches could affect the turgor pressure of the cells to prompt the fruit to enter the first stage of softening. Moreover, cell wall degradation served as the primary stimulus for the second phase of fruit softening [61]. Cell wall elements can be altered through sophisticated coordination during softening, with compositional transformations linked to the activity of cell wall decomposing enzymes, such as cellulase, polygalacturonase, β-galactosidase, and pectin methyl esterase [62,63,64]. Fruit cell wall polysaccharides contain approximately 20–35% cellulose [65]. Hence, the increase in cellulase activity is crucial in the softening process. 1-MCP can inhibit cellulase activity and reduce its gene expression by altering the gas composition around the fruit [66]. In our study, the cellulase activity of different kiwifruit varieties showed an increasing trend, indicating that with the extension of storage time, the cellulose in the cell walls was continuously hydrolyzed, thereby affecting the texture of the fruit. However, 1-MCP treatment inhibited cellulase activity in a concentration-dependent manner. Compared to the untreated group, the treatment of 1.0 μL/L 1-MCP exhibited a more stable impact on delaying the increase in cellulase activity across all kiwifruit varieties (Figure 1F). This aligns with the observed delay of fruit hardness reduction due to 1-MCP treatment, indicating that the treatment can preserve the cell wall structure by reducing the activity of cell-wall-degrading enzymes [67], which in turn could contribute to mitigating the decline in fruit hardness and the deterioration in kiwifruit quality.

#### 3.1.3. Effects of 1-MCP Treatment on Postharvest Antioxidant Levels in Kiwifruits

AsA is a characteristic attribute of the kiwifruit antioxidant quality, with most kiwifruit varieties experiencing a loss of AsA of up to 50–70% during storage [68]. The AsA content is the highest at the early stage of fruit development. After fruit ripening, the AsA level decreases and is then maintained at a stable level until full ripening [69]. According to gene expression analysis, 1-MCP positively regulates many AsA modification genes and candidate *bHLH* transcription factors. Additionally, upregulation of AsA biosynthesis genes and downregulation of degradation genes collectively contribute to the maintenance of AsA content in kiwifruit during storage [70]. In this research, the general pattern of AsA contents in kiwifruit subjected to different concentrations of 1-MCP exhibited a gradual decline with the extension of storage period. In particular, the AsA levels of ‘Xuxiang’ kiwifruit exposed to 1.0 μL/L 1-MCP exhibited an initial increase followed by a decrease in the prestorage period, possibly attributed to the ripening and accumulation impact of AsA in the harvested fruit. Furthermore, ‘Huayou’, which naturally had the highest AsA concentration, experienced a sharp decline and maintained a low level from 9 d to 12 d of storage, whereas the application of 1-MCP notably postponed the decline in AsA contents during this period (Figure 1G). These results reveal that 1-MCP can regulate fruit developmental processes and the stress tolerance by preserving AsA levels within kiwifruit [71].

The capacity of compounds in food to eliminate free radicals can demonstrate its antioxidant effect and capability to inhibit the initiation of the oxidation chain [72]. ABTS is a protonated radical exhibiting a characteristic peak at 734 nm, and the absorption value diminishes upon deprotonation during the transition to its nonradical state [73]. On the other hand, ·OH facilitates the specific cleavage of polysaccharides within the cell wall [74]. It has been discovered that 1-MCP treatment could impact the ABTS free radical scavenging capability through the upregulation of gene expression leading to enhanced phenolics accumulation, and that the higher the level of phenolic compounds in the plant, the stronger the free radical scavenging capacity [75,76,77]. In our study, the ABTS radical scavenging capacity of kiwifruit treated with different concentrations of 1-MCP and stored for 15 d showed an initial increase followed by a decrease. Under identical storage conditions, the ABTS radical scavenging ability of the ‘Xuxiang’ variety treated with 0.5 μL/L 1-MCP was notably superior compared to the other two varieties at the later storage phase. Meanwhile, the overall ABTS radical scavenging capacity of ‘Hayward’ was relatively lower, which was improved through treatment with 1.0 μL/L 1-MCP (Figure 1H). On the other hand, the ·OH radical scavenging capacity of ‘Hayward’ increased slightly with increasing concentration and did not change significantly in ‘Xuxiang’ (Figure 1I). Our research indicates that the declines in ABTS and ·OH radical scavenging abilities might be postponed following 1-MCP application on ‘Xuxiang’ and ‘Hayward’ varieties. But for ‘Huayou’, the scavenging ability for both radicals in 1-MCP-treated fruits was lower compared to the untreated group as storage time increased, which may be attributed to its higher AsA content [78]. Differences in the effects of 1-MCP on the free radical scavenging capacity of different kiwifruit cultivars are related to their varying antioxidant capacities, stemming from differences in the levels and types of phenolic compounds present in distinct cultivars and genotypes [79].

### 3.2. Metabolic Characteristics of Kiwifruit Postharvest Treated with 1-MCP

#### 3.2.1. Analytical Performances of Kiwifruit Metabolic Profiling

**Figure 2:**
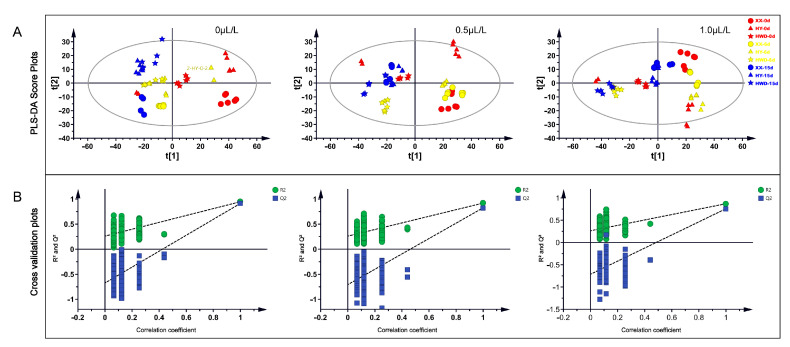
Multivariate analyses. (**A**) PLS-DA score plots of metabolites from different kiwifruit varieties. (**B**) The cross-validation plots of the corresponding PLS-DA modes with 200 times permutation tests. XX, HY, and HWD represent ‘Xuxiang’, ‘Huayou’, and ‘Hayward’, respectively.

Partial least squares discriminant analysis (PLS-DA) is a widely recognized method for extracting features and conducting discriminant analysis in the chemometrics field [80]. The impact of 1-MCP treatment on the kiwifruit storage metabolic profile can be depicted through PLS-DA distribution plots. In Figure 2A, kiwifruits untreated with 1-MCP exhibited distinct segregation trends on the first principal component at different storage stages (0 d, 6 d, and 15 d), while ‘Xuxiang’ demonstrated a clear trend of separation on the second principal component with the other two varieties (i.e., ‘Huayou’ and ‘Hayward’). After treatment with 0.5 μL/L 1-MCP, kiwifruits exhibited a marked tendency towards segregation on the second principal component as the storage period progressed. Furthermore, kiwifruits treated with 1.0 μL/L 1-MCP displayed a noticeable segregation trend on both the first and second principal components throughout the storage duration. The cross-validation graph (Figure 2B) results confirmed the reliabilities of the PLS-DA models.

#### 3.2.2. Identification and Clustering Analysis of Metabolites in Kiwifruits

**Figure 3:**
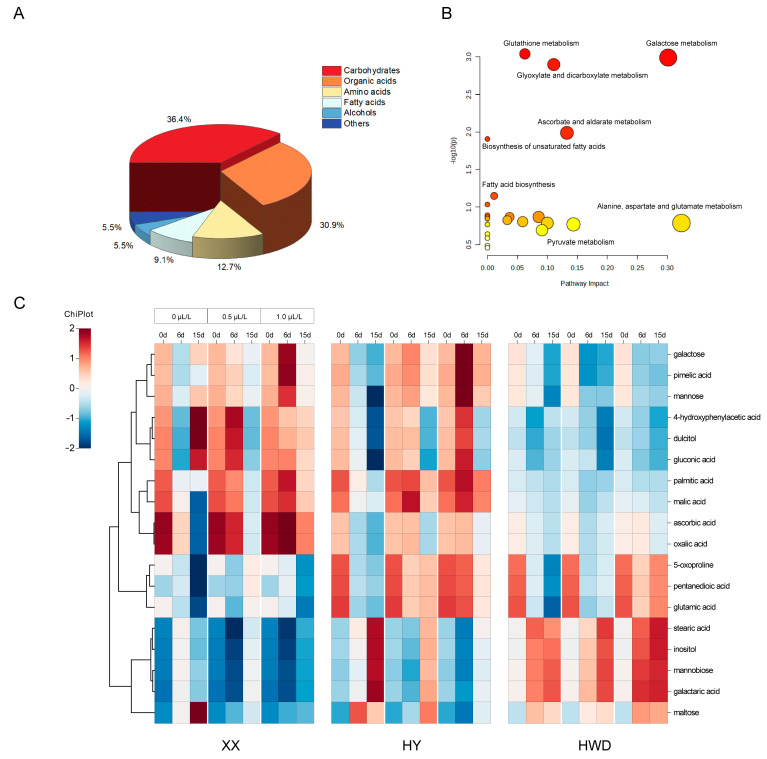
Effects of 1-MCP treatment on the metabolism of kiwifruits. (**A**) Distribution map of total metabolites categories, (**B**) metabolic pathway enrichment map, and (**C**) heatmap analysis of differential metabolites. In the heat map, red and blue represent the high and low relative contents of metabolites. XX, HY, and HWD represent ‘Xuxiang’, ‘Huayou’, and ‘Hayward’, respectively.

In this research, kiwifruits were examined using the GC–MS metabolomics method, which led to the identification of 55 metabolites. Among these, 20 were carbohydrates, making up approximately 36.4% of all metabolites. Additionally, 17 organic acids comprised 30.9% of the total metabolites, whereas seven amino acids and five fatty acids accounted for 12.7% and 9.1%, respectively (Figure 3A). In order to delve deeper into the dual impacts of varying concentrations of 1-MCP treatment and storage duration, a two-way ANOVA was employed to differentiate the metabolic repercussions of the two variables (cultivar and storage period) on the kiwifruit storage, and 18 compounds were screened as significantly different metabolites (*p* < 0.05). We used MetaboAnalyst (http://www.metaboanalyst.ca/MetaboAnalyst/ (accessed on 4 June 2024)) to contrast the enrichment of metabolic pathways based on differential metabolites (Figure 3B). The enrichment analysis indicated that the main metabolites were involved in pathways such as glutathione metabolism; alanine, aspartate, and glutamate metabolism; galactose metabolism; and ascorbic acid and aldolase metabolism. Cluster analysis of different metabolites was conducted using Chiplot (https://www.chiplot.online/ (accessed on 4 June 2024)) to intuitively reveal the alterations in metabolite contents of different kiwifruit varieties (Figure 3C). Changes of carbohydrates during fruit ripening can provide the energy foundation for cellular metabolism, and a high level of carbohydrates helps to preserve the kiwifruit sweetness during the later stages of storage. Our research found that during the initial storage period, the levels of monosaccharides such as galactose and mannose in ‘Xuxiang’ and ‘Huayou’ were higher than that in ‘Hayward’. Conversely, ‘Hayward’ had higher contents of disaccharides (e.g., maltose and mannobiose) compared to the other two varieties. Additionally, in the conversion processes of certain saccharic acids (e.g., galactaric acid, gluconic acid), ‘Hayward’ also exhibited a trend opposite to the other two varieties. These differences may arise from carbohydrate partitioning mechanisms during fruit development in fruit trees coordinating the plant’s supply–demand equilibrium in a species-specific manner, which in turn affect the accumulation of fruit metabolites [59,81]. Moreover, our research indicated that the ‘Huayou’ kiwifruit treated with 0.5 μL/L 1-MCP accumulated higher contents of carbohydrates (such as galactose and mannose) compared to the group treated with 1.0 μL/L 1-MCP, suggesting that 0.5 μL/L 1-MCP treatment concentration was more effective in preserving the freshness of the ‘Huayou’ cultivar, which was supported by the quality index analysis. ‘Hayward’ kiwifruit showed increased accumulation of sugars (e.g., maltose and galacturonic acid) after 1-MCP treatment during the later storage (15 d), with higher treatment concentrations of 1-MCP leading to greater accumulation of these compounds, which was consistent with the results of the reducing sugar content analysis in the quality assessment. The above results show that the ripening mechanisms of various kiwifruit cultivars might be regulated by distinct carbohydrates, leading to flavor variations. Moreover, carbohydrate metabolism in kiwifruit exhibited notable alterations following 1-MCP treatment. This effect likely arose from 1-MCP’s ability to impede ethylene synthesis by obstructing its binding site [82], on account of prior research indicating a correlation between genes involved in starch breakdown in fruits and ethylene production [83]. Consequently, 1-MCP may induce fluctuations in the expression of specific genes associated with carbohydrate metabolism [84].

Amino acids and fatty acids are significant indicators of fruit ripeness and flavor quality, serving as precursors for the synthesis of volatiles, which can influence ethylene production and the nutritional quality of kiwifruit [85,86]. Amino acids, crucial nitrogen reserves in plants, are vital components of plant primary metabolism [87]. Our study observed a general decreasing trend in amino acid levels across three kiwifruit varieties (‘Xuxiang’, ‘Huayou’, and ‘Hayward’); among them, ‘Xuxiang’ kiwifruit consistently exhibited lower levels of amino acid (e.g., glutamate, pyroglutamate) compared to the other two varieties. Additionally, the amino acids contents of all kiwifruit varieties treated with 1-MCP were higher than those of the untreated group during mid- and later storage stages. Glutamate, as a plant defense signal, can be actively released or leaked from damaged cells when the plant is injured, to trigger plant Ca^2+^ signaling and activate the plant defense response [88]. Additionally, glutamic acid was also a precursor for the synthesis of several other amino acids (proline, ornithine, and arginine), which play important roles in the syntheses of stress metabolites [89,90]. Therefore, our study found that the elevated levels of amino acids resulting from 1-MCP treatment could indicate improved disease resistance in fruits, ultimately prolonging their storage lifespan.

Fatty acids, as essential constituents of lipids, performed crucial functions in the fruits ripening and the flavor formation [91], and significant alterations in fatty acids composition among various kiwifruit cultivars have been found [92]. Palmitic and stearic acids are the most prevalent fatty acids in plants. Our study observed that the increases in stearic acid and the decrease in palmitic acid in ‘Xuxiang’ and ‘Huayou’ kiwifruits were delayed after 1-MCP treatment. Conversely, a higher content of stearic acid was found in 1-MCP-treated ‘Hayward’ kiwifruit. These results indicate that the impacts of 1-MCP treatment on kiwifruit fatty acids contents vary by variety. Furthermore, the fatty acid composition played a crucial role in maintaining cellular homeostasis, which in turn helped preserve membrane integrity during kiwifruit storage and prevented fruit softening [93].

#### 3.2.3. Correlation Analysis

**Figure 4:**
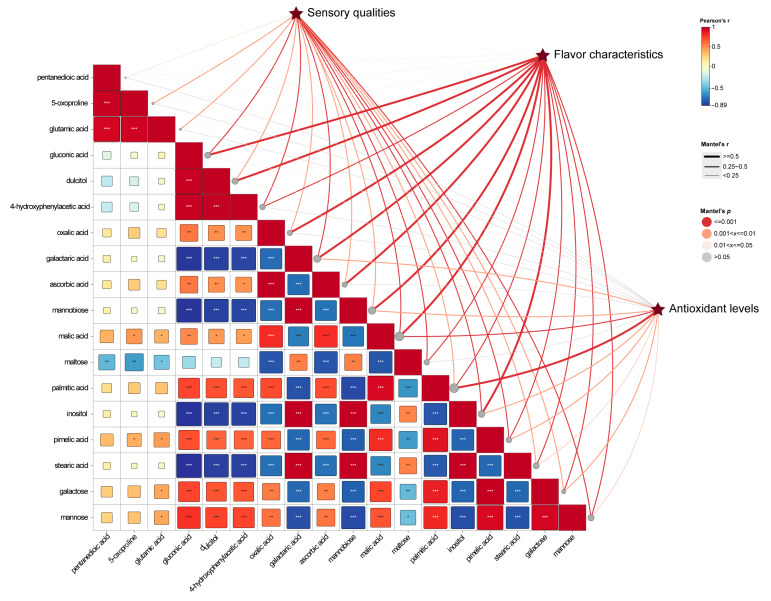
Correlation analysis. The rectangles reflect the Pearson correlation coefficients between various metabolites, colored from negative correlation (blue) to positive correlation (red) based on the magnitude. The range of *p*-values for metabolites was shown with symbols (*: *p* < 0.05, **: *p* < 0.01, ***: *p* < 0.001). The connecting lines illustrate the associations between physiological indicators and metabolites based on Mantel test, with thicker lines representing stronger correlations and thinner lines indicating weaker ones. The color of the lines reflects the magnitude of the *p*-values.

The Mantel correlation coefficient analysis (https://www.chiplot.online/ accessed on 12 June 2024) was applied to investigate the relationships between kiwifruit quality attributes (i.e., sensory, flavor, antioxidant) and differential metabolites throughout the storage period (Figure 4), revealing strong correlations among the majority of the differential compounds with kiwifruit flavor profiles and weak correlations with antioxidants. Among them, the compounds that were significantly correlated (r ≥ 0.5, *p* < 0.001) with flavor characteristics included gluconic acid, galacturonic acid, oxalic acid, mannobiose, and myoinositol; and palmitic acid was significantly associated with antioxidant levels. In addition, most sugars and saccharic acids were correlated with fruit sensory quality, flavor characteristics, and antioxidant levels, indicating that sugar metabolism had the most significant effect on fruit physiology during kiwifruit storage. In contrast, the correlation between amino acids and fruit quality attributes was weak.

On the other hand, the relationships between individual metabolites were analyzed using Pearson’s correlation coefficient. It was observed that monosaccharides like mannose and galactose were positively correlated with palmitic acid and malic acid, and negatively correlated with stearic acid, disaccharides, and saccharic acid. Fatty acids such as palmitic and stearic acids exhibited opposite correlations with other metabolites, indicating that their functions in kiwifruit metabolism may be different.

## 4. Conclusions

Physiological–biochemical methods and metabolomics technology were combined to study the effects of different concentrations of 1-MCP (0 μL/L, 0.5 μL/L, 1.0 μL/L) on postharvest quality and small molecular metabolites of three important commercial kiwifruit varieties (i.e., ‘Xuxiang’, ‘Huayou’, and ‘Hayward’), which are extensively cultivated around the world. This study revealed that 1.0 μL/L 1-MCP had a more pronounced preservation effect on ‘Xuxiang’ and ‘Hayward’ varieties, resulting in a notable delay in the decrease in fruit hardness, the increases in ripening index and cellulase activity, and the inhibition of AsA content reduction. On the other hand, 0.5 μL/L 1-MCP demonstrated a stronger impact on chlorophyll level and ripening index of ‘Huayou’, showing the most effective preservation effect. Furthermore, 1-MCP treatment can also significantly influence carbohydrate metabolism, fatty acid metabolism, and amino acid metabolism in different kiwifruit varieties. Correlation analysis highlighted that sugar metabolism was closely associated with postharvest physiological quality. This study analyzed the molecular mechanism of 1-MCP in regulating the quality characteristics of various kiwifruit varieties at physiological and small molecule metabolism levels. These findings may offer new targets for the development of kiwifruits’ storage and preservation technology.

## Data Availability

The original contributions presented in the study are included in the article/Supplementary Material, further inquiries can be directed to the corresponding author.

## Supplementary Materials

The following supporting information can be downloaded at: https://www.mdpi.com/article/10.3390/foods13223632/s1, Figure S1: The increase rates of physiological indexes of kiwifruit varieties at 15 d compared with that at 0 d after 1-MCP treatment; Figure S2: Effects of 1-MCP treatment on (A) SSC and (B) TA in postharvest kiwifruits. XX, HY and HWD represent kiwifruit varieties ‘Xuxiang’, ‘Huayou’ and ‘Hayward’, respectively.
