# Supplementary material for: Effects of 1-Methylcyclopropene Treatment on Postharvest Quality and Metabolism of Different Kiwifruit Varieties
Source: Foods. 2024 Nov 14;13(22):3632. doi: 10.3390/foods13223632 (PMC11593305; doi:10.3390/foods13223632)
Supplement: Supplementary file 1 [file foods-13-03632-s001.zip › foods-3303753-supplementary.pdf]

Effects of 1-methylcyclopropene treatment on postharvest quality and metabolism of different kiwifruit varieties

Yanni Zhao[1,2], Meiru Yan[1], Kun Zhang[1], Xuan Wu[1], Zi Wang[1], Ting Shao[1], Jing Lei[3], Xuefeng Chen[1,2], Huan Liu[1,2*]

[1]School of Food and Biological Engineering, Shaanxi University of Science & Technology, Xi'an 710021, China

[2]Shaanxi Research Institute of Agricultural Products Processing Technology, Xi'an 710021, China

[3]Shaanxi Rural Science and Technology Development Center, Xi'an 710054, China

[*]Corresponding authors:

Huan Liu Email: liuhuan@sust.edu.cn, Tel and Fax: +86-029-86168589

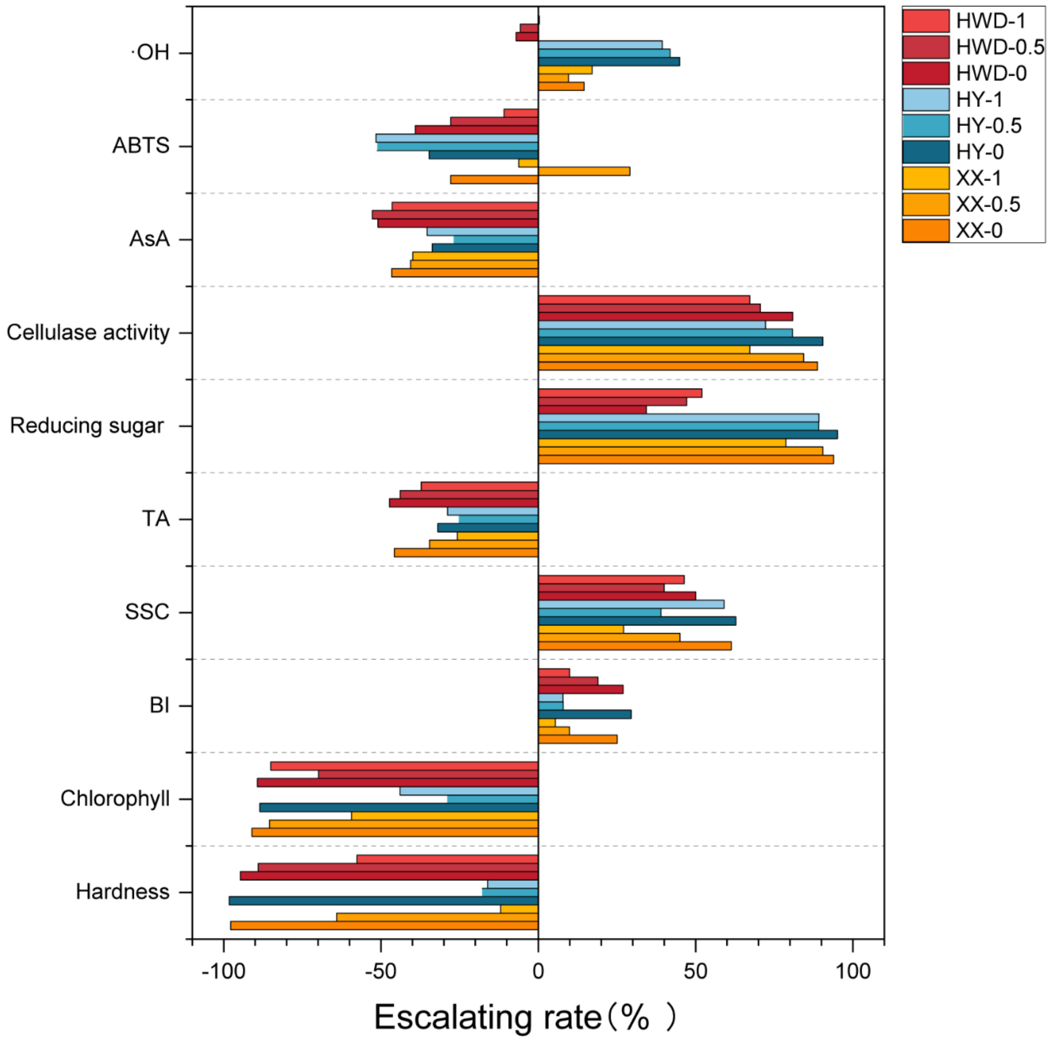

Fig. S1. The increase rates of physiological indexes of kiwifruit varieties at 15 d compared with that at 0 d after 1-MCP treatment.

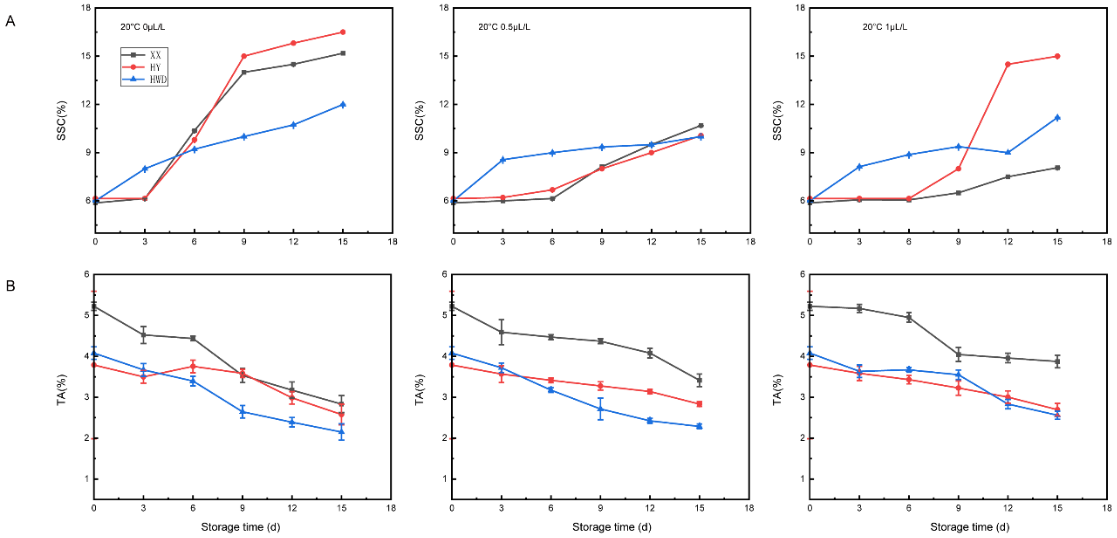

Fig. S2. Effects of 1-MCP treatment on (A) SSC and (B) TA in postharvest kiwifruits. XX, HY and HWD represent kiwifruit varieties ‘Xuxiang’, ‘Huayou’ and ‘Hayward’, respectively.
